# Global declines in human‐driven mangrove loss

**DOI:** 10.1111/gcb.15275

**Published:** 2020-08-03

**Authors:** Liza Goldberg, David Lagomasino, Nathan Thomas, Temilola Fatoyinbo

**Affiliations:** ^1^ Atholton High School Columbia MD USA; ^2^ Biospheric Sciences Laboratory NASA Goddard Space Flight Center Greenbelt MD USA; ^3^ Earth Systems Science Interdisciplinary Center University of Maryland College Park MD USA; ^4^ Department of Coastal Studies East Carolina University Wanchese NC USA

**Keywords:** climate change, commodities, deforestation, Landsat, mangrove, shoreline erosion

## Abstract

Global mangrove loss has been attributed primarily to human activity. Anthropogenic loss hotspots across Southeast Asia and around the world have characterized the ecosystem as highly threatened, though natural processes such as erosion can also play a significant role in forest vulnerability. However, the extent of human and natural threats has not been fully quantified at the global scale. Here, using a Random Forest‐based analysis of over one million Landsat images, we present the first 30 m resolution global maps of the drivers of mangrove loss from 2000 to 2016, capturing both human‐driven and natural stressors. We estimate that 62% of global losses between 2000 and 2016 resulted from land‐use change, primarily through conversion to aquaculture and agriculture. Up to 80% of these human‐driven losses occurred within six Southeast Asian nations, reflecting the regional emphasis on enhancing aquaculture for export to support economic development. Both anthropogenic and natural losses declined between 2000 and 2016, though slower declines in natural loss caused an increase in their relative contribution to total global loss area. We attribute the decline in anthropogenic losses to the regionally dependent combination of increased emphasis on conservation efforts and a lack of remaining mangroves viable for conversion. While efforts to restore and protect mangroves appear to be effective over decadal timescales, the emergence of natural drivers of loss presents an immediate challenge for coastal adaptation. We anticipate that our results will inform decision‐making within conservation and restoration initiatives by providing a locally relevant understanding of the causes of mangrove loss.

## Graphical abstract

Here we provide the first global‐scale, high‐resolution assessment of the anthropogenic and natural drivers of mangrove losses from 2000 to 2016. We show that loss rates have declined by 73% since 2000, particularly as conversion of mangroves to aquaculture, agriculture, and settlement decreases globally. We demonstrate that future mangrove resilience will depend primarily on the progression of sea level rise and extreme events, especially in highly populated coastal regions.

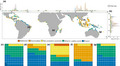

## 1 INTRODUCTION

Mangrove forests are globally recognized as highly carbon‐rich tropical ecosystems that provide a range of critical economic and ecological services to surrounding coastal populations (Barbier et al., 2011; Donato et al., 2011). However, mangroves have been heavily impacted by degradation and deforestation, with 20%–35% of global mangrove extent lost over the last 50 years (Polidoro et al., 2010). Twentieth‐century mangrove losses were largely dominated by forest clearing and exploitation for timber production and raw materials, as well as rapid coastal population growth and urban expansion (Richards & Friess, 2016; Thomas et al., 2017). Economic and political emphasis on aquaculture development has led to large‐scale conversion of mangroves to shrimp and rice aquaculture ponds to take advantage of the growth in global aquaculture demand (Friess et al., 2016). Climate change and oceanic warming are expected to increase global sea levels, wave energy (Reguero, Losada, & Méndez, 2019) and the intensity and frequency of extreme weather events (EWE) such as droughts and tropical cyclones (Bhatia, Vecchi, Murakami, Underwood, & Kossin, 2018; Murakami et al., 2020), exacerbating these large‐scale losses from land‐use change (Thomas et al., 2017). Measuring the impacts of humans and natural processes on these ecosystems will be critical to the advancement of Blue Carbon science and policy (Macreadie et al., 2019).

Mangrove forest extent and change has been mapped at high resolution from remotely sensed data (Bunting et al., 2018; Giri et al., 2011; Hamilton & Casey, 2016), providing an understanding of the global spatial distribution of mangroves and their rates of change over decadal timescales. Recent remote sensing‐based datasets documenting global mangrove deforestation have yielded annual loss rates of between 0.26 and 0.66 percent loss per year (Hamilton & Casey, 2016). These datasets have further been combined with field data to map higher‐level mangrove attributes at regional and global scales, including mangrove height (Simard et al., 2019), aboveground biomass/carbon (Simard et al., 2019; Tang et al., 2018) and soil carbon (Atwood et al., 2017; Jardine & Siikamäki, 2014; Rovai et al., 2018; Sanderman et al., 2018), and forest fragmentation (Bryan‐Brown et al., 2020). These studies highlight the important role of remote sensing in the monitoring of regional and global trends in mangrove ecosystem health and change.

Understanding the causes of mangrove loss is important for establishing opportunities for blue carbon projects (Macreadie et al., 2019). In particular, quantifying the reasons for mangrove loss is critical towards estimation of carbon emissions (López‐Angarita, Tilley, Hawkins, Pedraza, & Roberts, 2018; Sasmito et al., 2019), and the opportunities for enhancing blue carbon through management (López‐Angarita et al., 2018). Using two‐high resolution datasets on mangrove extent (Giri et al., 2011) and global deforestation (Hansen et al., 2013), mangrove loss in Southeast Asia was primarily associated with anthropogenic land conversion to agriculture and aquaculture (Richards & Friess, 2016). More recently, a coarse‐scale, qualitative global assessment of the distribution of natural and anthropogenic causes of mangrove loss identified the broad influence of natural losses across 20% of 1,168 tiles intersecting global mangrove regions, along with similar hotspots of anthropogenic change recorded for Southeast Asia (Thomas et al. 2017). These studies helped to identify that the primary drivers of mangrove loss are unequally distributed around the world. However, to date, there has not been a global quantitative assessment of both the human and natural drivers of mangrove loss at the global scale, which is required to facilitate carbon mitigation strategies (Taillardat, Friess, & Lupascu, 2018) and mangrove conservation (Romañach et al., 2018). Therefore, spatially explicit information is needed to identify the prevalence and variety of anthropogenic stressors driving forest vulnerability at local scales. Gains in mangrove area have also become increasingly prevalent in some regions, helping to offset losses (Hakimdavar et al., 2020; Lagomasino et al., 2019), though identifying the causes of loss can address continued threats necessary to move forward toward zero net loss in global mangroves.

Here we present the first global, high‐resolution, mangrove‐specific land use change models, capturing the broad range of human‐driven stressors and natural disturbances that occur along the coastal margin. We used Normalized Difference Vegetation Index (NDVI)‐based anomalies to identify regions of mangrove loss from 2000 to 2005, 2005 to 2010, and 2010 to 2016, using Landsat 5, 7, and 8 archive data at a scale of 30 m. Random Forest machine learning algorithms were then employed to classify mangrove land cover changes using a pixel‐based approach that quantified wet pixels, dry pixels, and water pixels (Figure S1). Lastly, the land cover maps were passed through a series of decision trees to separate anthropogenic and natural losses (Figure S2). We ultimately produce global 30 m resolution loss extent, land cover change, and loss driver maps with uncertainties for all mangrove‐holding nations from 2000 to 2005 (*loss2005*), 2005 to 2010 (*loss2010*), and 2010 to 2016 (*loss2016*).

## 2 MATERIALS AND METHODS

### 2.1 Loss extent mapping

A Landsat‐based NDVI anomaly algorithm was used to aggregate changes in mangrove greenness over time, identifying pixels of loss. A reference period was designated using the median NDVI value from January 1998 through December 2001 covering Landsat 5 TM and Landsat 7 ETM+ images (Supplemental Methods: Figure S4). Individual pixel stacks that did not have at least 10 quality pixels were excluded from the analysis. The reference NDVI for the period 1998–2001 was then subtracted from each of the images in the observation period, which ranged from January 2001 through December 2016 as outlined in Lagomasino et al. (2019). Summing the difference from the reference period in each overlapping pixel stack produced a cumulative anomaly. These anomaly values were also normalized for the total number of images with non‐null values for individual pixels, resulting in a mean change in NDVI over the observation period when compared to the reference. We selected a change threshold of −0.2 based on Lagomasino et al. (2019) that occurred within the Global Mangrove Forests Dataset (GMFD; Giri et al., 2011) to be considered a permanent loss of mangroves. In order to determine the accleration or deceleration of mangrove loss, we repeated this analysis using temporal subsets: 2000–2005, 2005–2010, and 2010–2016. For each subset, loss was only expected to occur within the larger 2000–2016 total loss extent.

### 2.2 Land cover change classification

We used a machine learning‐based classification approach to map land cover changes in all loss pixels, overcoming the potential subjectivity and bias associated with previous studies that relied on manual classification of primary drivers in each unit area (Thomas et al., 2017). The remote sensing analysis was completed end‐to‐end in Google Earth Engine, on account of its capacity to process large volumes of global‐scale predictor Landsat data (Gorelick et al., 2017).

Using high‐resolution imagery from Google Earth, we categorized the land cover type of three training points in each of 1,168 1°×1° grid cells containing mangrove forest in the year 2000. Points were delegated into one of three initial classes as follows: wet soil, dry soil, and water, based on their dominant visual characteristics (Figure S5). Circular polygons of radius one hectare were generated around each point to increase the total number of pixels sampled in each class. The comprehensive training set included 1,137 water polygons, 771 agricultural polygons, and 761 urban polygons; this range of available training points in each class resulted from differing availabilities of each land cover type within the global grid cells sampled.

A 2014–2018 Landsat 7 and 8 composite was generated in order to provide predictor data for the conditions at all training points at the end of the study period. The CFMask cloud‐removal algorithm eliminated cloud shadows and clouds from all Landsat imagery. Values for each of 7 predictor variables—NDVI, normalized difference moisture index, modified normalized difference water index, green chlorophyll vegetation index, surface reflectance, ratio54, and ratio35—were generated for each training point. We selected a random forest classifier to run the final global classifications, owing to its comparatively high performance on global classification problems (James, Witten, Hastie, & Tibshirani, 2000). A single global classification of the three land cover types was generated for the period 2014–2018, capturing land cover change from mangroves in 2000 to other land covers in 2016.

### 2.3 Land use change mapping

The results of the land cover mapping were then used to classify the losses into specific land use change categories (Figure S6). Because of the similarity in spectral signatures that could occur among land cover classes, we could not distinguish land use changes solely on the basis of spectral behavior. For example, the vastly different land uses/land covers of aquaculture and coastal waters share similar spectral characteristics of water, as do agriculture and mudflats within the wet soils land cover class, and impervious surfaces and sand within the dry soils class. Therefore, Landsat data alone could not separate between land use types. To distinguish each ultimate land use, a recursive partition model (commonly known as a decision‐tree model) used several open‐source datasets to separate each land cover category into one of five loss driver categories: erosion , commodities , settlement , non‐productive conversion, and extreme weather events. Each pixel of land cover change passed through a unique decision tree based on its initial land cover category until it reached its terminal loss driver assignment (Figure S2). See Supplemental Methods for a detailed description of the land use decision tree modeling.

In combining the extent of loss from each individual driver, we produced a cumulative global land use change map for 2000–2016. We separated losses into their individual epochs—2000 to 2005, 2005 to 2010, and 2010 to 2016—by cutting the loss driver maps with each epoch's respective loss extent map. Thus, no loss driver maps were regenerated on an individual epoch basis, because of the unlikelihood of major shifts in post‐conversion land use in the 16 year period. The cumulative dataset consists of 30 m resolution loss extent, land cover change, and loss driver maps for each epoch.

### 2.4 Validation

We assessed the accuracy of our land use change maps and estimated the uncertainty of area measurements using recent best practices (Olofsson et al., 2014). To assess the accuracy of the mangrove loss drivers, we randomly generated a total of 2,476 validation points for the entire globe that were allocated based on the proportion of each land cover class: 1,104 for commodities, 522 for erosion, 384 for non‐productive conversion, 266 for extreme weather events, and 200 for human settlement (Figure S7). Each point was allocated within our loss extent map, in order to solely determine the accuracy of the loss driver maps. However, in only sampling within our designated loss extent maps, we exclude loss omission error from our accuracy assessment.

The class assignment of each validation point was evaluated using a RSGISLib‐based QGIS class accuracy plugin that enabled reference to the most recent Google Earth imagery in all locations (Bunting, Clewley, Lucas, & Gillingham, 2014). An error matrix was derived for the 2,476 sample counts calculating the producer's accuracy, user's accuracy, and an overall accuracy (Table S2). We then calculated the variance for each of the accuracies to estimate the 95% confidence interval. Using the original area proportions for each class and the estimated area proportions from the reference data, we then derived a reference‐corrected estimate of the total areas for each mangrove loss driver. A 95% confidence interval for the area of each class was calculated using the standard error for each estimated mangrove loss driver class (Olofsson et al., 2014). Overall map accuracy was 81.5%. The user's accuracies for each class were 88.3% for commodities, 81.6% for erosion, 77.5% for settlement, 72.3% for non‐productive conversion, and 68.1% for extreme weather events. Producer's accuracies for each class were 89.0% for commodities, 85.2% for settlement, 78.0% for extreme weather events, 75.5% for erosion, and 74.6% for non‐productive conversion (Table S2).

## 3 RESULTS

**FIGURE 1:**
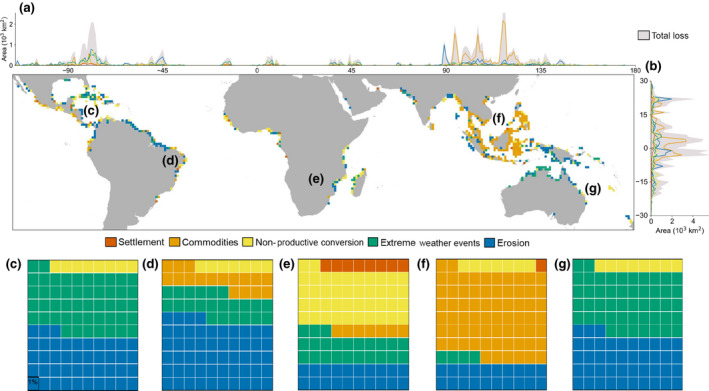
Global distribution of mangrove loss and its drivers. (a) The longitudinal distribution of total mangrove loss and the relative contribution of its primary drivers. Different colors represent unique drivers of mangrove loss. (b) The latitudinal distribution of total mangrove loss and the relative contribution of its primary drivers. (c‐g) Global distribution of mangrove loss and associated drivers from 2000 to 2016 at 1°×1° resolution, with the relative contribution (percentage) of primary drivers per continent: (c) North America, (d) South America, (e) Africa, (f) Asia, (g) Australia together with Oceania.

Overall, 3,363 km^2^ (2.1%) of global mangrove area was lost between 2000 and 2016, at an average annual rate of 0.13%. Human activity persisted as the dominant agent of mangrove loss but was unevenly distributed around the world. From 2000 to 2016, anthropogenic causes accounted for 62% of total global mangrove loss area (Figure 1). Commodities (CM), a combination of rice, shrimp, and oil palm cultivation, served as the primary global driver of mangrove loss, constituting 47% (1,596 ± 42 km^2^) of global losses from 2000 to 2016. Non‐productive conversions (NPC) caused 12% (398 ± 29 km^2^) of global losses, with reclaimed lands for human settlements (ST) only representing 3% (96 ± 15 km^2^). The remaining 38% of total mangrove loss was attributed to natural causes. Shoreline erosion (ER) represented the second highest percentage of global losses at 27% (912 ± 41 km^2^) and extreme weather events (EWE) contributed 11% of losses (361 ± 31 km^2^).

### 3.1 Anthropogenic losses

**FIGURE 2:**
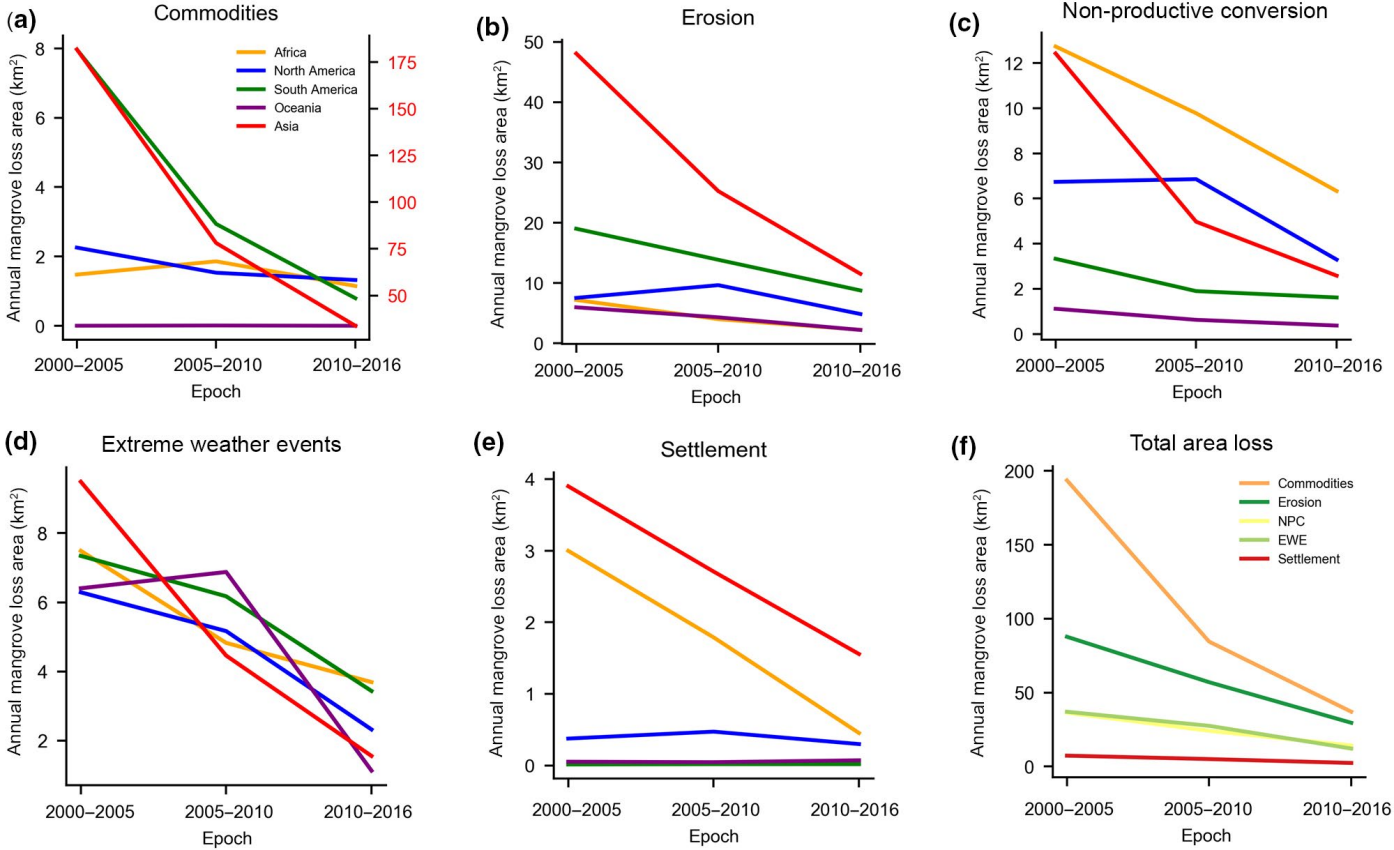
Annual mangrove loss rates by driver and epoch. Mangrove loss rates were calculated by dividing the total mangrove area lost to each driver per epoch by the number of years in the epoch. Panels (a–e) show mangrove loss rates by continent, while panel (f) shows global total loss rates by driver. EWE represents loss by extreme weather events, and NPC represents loss by non‐productive conversion

Human activity was the primary agent of change between the nominal dates of 2000 and 2016. However, the area of mangroves converted by direct human intervention (e.g., CR, NPC, and ST) declined by 73% over the 16 year period. Approximately 1,186 km^2^ were lost in the first epoch (*loss2005*), decreasing to 314 km^2^ in the last epoch (*loss2016*; Figure 2). Similarly, the percentage of total mangrove loss attributed by human impacts decreased by 10% over the same period, from a high of 66% in *loss2005* to a low of 56% in *loss2016*.

**FIGURE 3:**
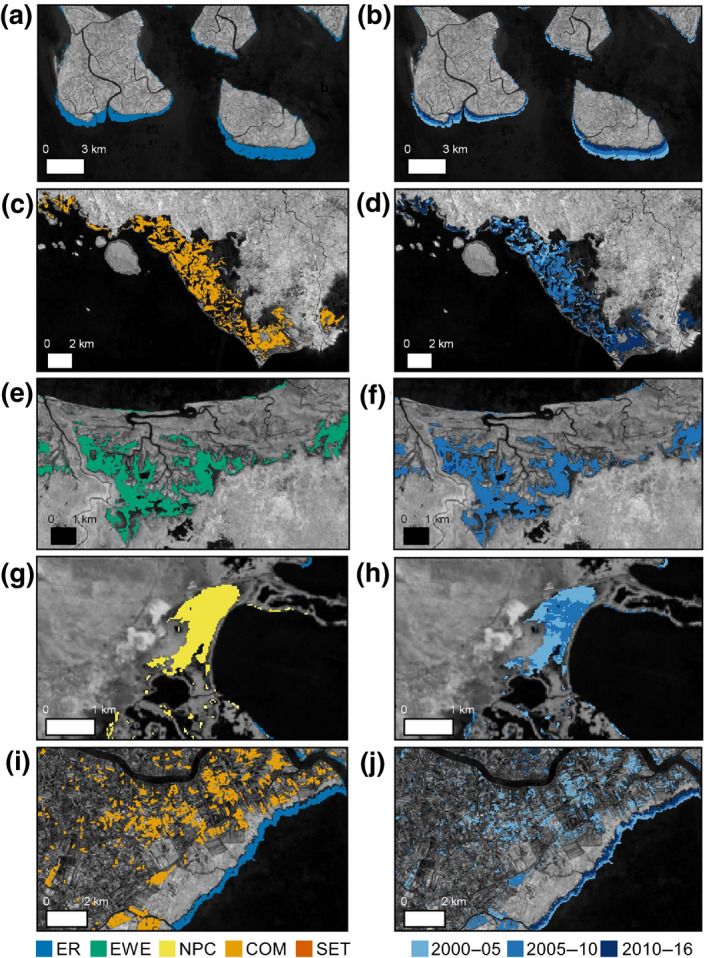
Local heterogeneity in mangrove loss drivers. The left image shows the 2000–2016 loss driver map, and the right image shows the epoch of loss. (a, b) Erosional banding in the Bangladesh Sundarbans. (c, d) Conversion to commodities in Tanjung Panjang Nature Reserve in Sulawesi, Indonesia. (e, f) Conversion by extreme weather events resulting from Cyclone Guba in Oro Province, Papua New Guinea. (g, h) Non‐productive conversion from hydrologic disturbance following construction of Rocky Point Main Road near Colon Bay, Cuba. (i, j) Simultaneous ER and conversion to CM in the Mekong Delta, Vietnam. COM, conversion to commodities; ER, erosion, EWE, extreme weather events; NPC, non‐productive conversion; SET, settlement‐driven loss

The vast majority of the total global direct anthropogenic loss, nearly 80% (2,068 km^2^, was concentrated in just six nations: Indonesia, Myanmar, Malaysia, the Philippines, Thailand, and Vietnam (Dataset S1). Within these six countries, 82% of loss was human driven, compared to only 33% in all other nations. Even as anthropogenic losses declined globally, they remained consistently concentrated in Southeast Asia primarily due to widespread mangrove conversion to aquaculture and agriculture. Approximately 92% of all global CM losses occurred in Southeast Asia, serving as the primary loss driver in the majority (7 of 10) of the region's mangrove holding nations (Figure 1; Dataset S1). Although CM conversions were widely distributed throughout Southeast Asia, distinct hotspots were particularly prevalent in the Kalimantan and Sulawesi regions of Indonesia, the Mekong Delta in Vietnam and Rakhine state of Myanmar (Figure 3c,d; Figure S3). Of all anthropogenic drivers, commodity‐driven losses declined most substantially from 2000 to 2016, with a 77% decrease in total loss area (Figure 2).

Non‐productive conversion of mangroves constituted 398 km^2^ (12%) of global losses, with Africa remaining the only continent with NPC as the primary cause of loss (Figure 1f). At the country level, NPC represented more than half of national losses in 11 of the 22 African mangrove‐holding nations that experienced loss (Dataset S1). Petroleum extraction in the Niger Delta alone represented 20 km^2^ of NPC losses. Notable resource mining activities in other regions also included 5 km^2^ of NPC loss from Grasberg Mine tailings in Papua, Indonesia (Alonzo, Van Den Hoek, & Ahmed, 2016) and widespread hotspots of open pit coal mining in the Mahakam River of East Kalimantan, Indonesia (Toumbourou, Muhdar, Werner, & Bebbington, 2020). NPC‐driven losses ultimately declined by 46% from 268 km^2^ in *loss2005* to 129 km^2^ in *loss2016* (Figure 2c).

The conversion of mangrove forests to human settlement (ST) contributed least to global losses, at just 3% (96 ± 15 km^2^) of global loss extent. Rapid urban expansion into adjacent mangrove forests occurred in Ho Chi Minh City, Vietnam; Bangkok, Thailand; Lagos, Nigeria; and Conakry, Guinea. Settlement‐driven loss in all other nations remained below 3 km^2^ from 2000 to 2016. As with all other drivers, the proportion of human settlement‐driven loss declined by 65% from *loss2005* to *loss2016* (Figure 2e).

### 3.2 Natural losses

Natural losses of mangrove forests through shoreline ER and EWE remained pervasive throughout the world. Nearly all mangrove‐holding nations were affected by ER and/or EWE (Figure 1). Over the 16 year period, the total loss area attributed to natural causes (e.g., ER and EWE) declined from 624 km^2^ in *loss2005* to 249 km^2^ in *loss2016*. The decreasing rate of natural loss was significantly less than the declining rate of anthropogenic loss; thus, the relative contribution of natural drivers to global mangrove losses increased by 10% over the 16 year period, rising from just 34% (412 km^2^) of total losses in *loss2005* to 44% (195 km^2^) in *loss2016*. However, excluding the six nations overwhelmingly dominated by CM losses (>80%), natural losses from ER and EWE rose from 48% of total losses in *loss2005* to 68% in *loss2016*. ER was the primary cause of loss outside these six nations as well, representing nearly half (43%) of total losses from 2000 to 2016.

The most significant hotspot of mangrove ER loss occurred in Bangladesh, where loss along the seaward edge of the Sundarbans led to ER contributing nearly 80% of national losses (Table 1). Nearly 130 km^2^ of coastal ER also occurred along the eastern coast of Brazil, largely as a result of significant Amazon River discharge (Jahfer, Vinayachandran, & Nanjundiah, 2017). In some ER hotspots, natural losses adjacent to inland barriers such as ST and shrimp/rice ponds compounded loss rates, as mangrove forests became squeezed between development and the ocean, especially in the CM‐dominated hotspots of Southeast Asia. The pairing of ER and CM‐driven losses has led to substantial coastal squeeze in Vietnam's Mekong Delta and Indonesia's Mahakam Delta in particular, as ER has increasingly worn away the thin band of mangrove forest separating rice and shrimp ponds from the ocean (Figure 3i,j).

**TABLE 1:** Countries of most significant loss in each driver class. Of the top 15 mangrove‐holding nations (Giri et al. 2011), the five countries with the highest percent loss per class from 2000 to 2016

Country	Primary loss driver	Loss rate for primary driver 2000–2005 (%)	Loss rate for primary driver 2005–2010 (%)	Loss rate for primary driver 2010–2016 (%)
Myanmar (Burma)	Commodities	86.1	90.0	88.5
Guinea Bissau	Non‐productive conversion	56.2	59.6	45.7
Madagascar	Settlement	0.1	0.2	0.0
Bangladesh	Erosion	78.4	78.3	87.0
Australia	Extreme weather events	71.7	75.0	52.3

Losses from EWE such as cyclones, droughts, heatwaves, or extreme floods were evenly distributed across the globe, as EWE drove between 18% and 22% of the total loss area in each continent. EWE‐driven mangrove diebacks contributed the highest percentage of continental losses in Oceania, constituting nearly half of all loss from 2000 to 2016. Within Oceania, much of this loss occurred in the Oro Province of Papua New Guinea due to Cyclone Guba in November 2007 (Figure 3e,f). Frequent hurricane activity across Cuba caused extensive losses throughout the country, where 14% of global EWE‐driven loss occurred despite Cuba's comparatively low total loss area (Dataset S1). Smaller scale permanent losses attributed to EWE were observed in Florida's Everglades National Park (Lagomasino et al., 2020), Sundarbans, Bangladesh (Hazra, Ghosh, Dasgupta, & Sen, 2002), and Bay Islands, Honduras (Cahoon et al., 2003). Substantial EWE‐driven loss in the Gulf of Carpentaria, Northern Australia also coincided with climatic extremes such as drought and anomalously high temperatures in the region during that time (Duke et al., 2017).

## 4 DISCUSSION

### 4.1 The human footprint on global mangrove loss

**FIGURE 4:**
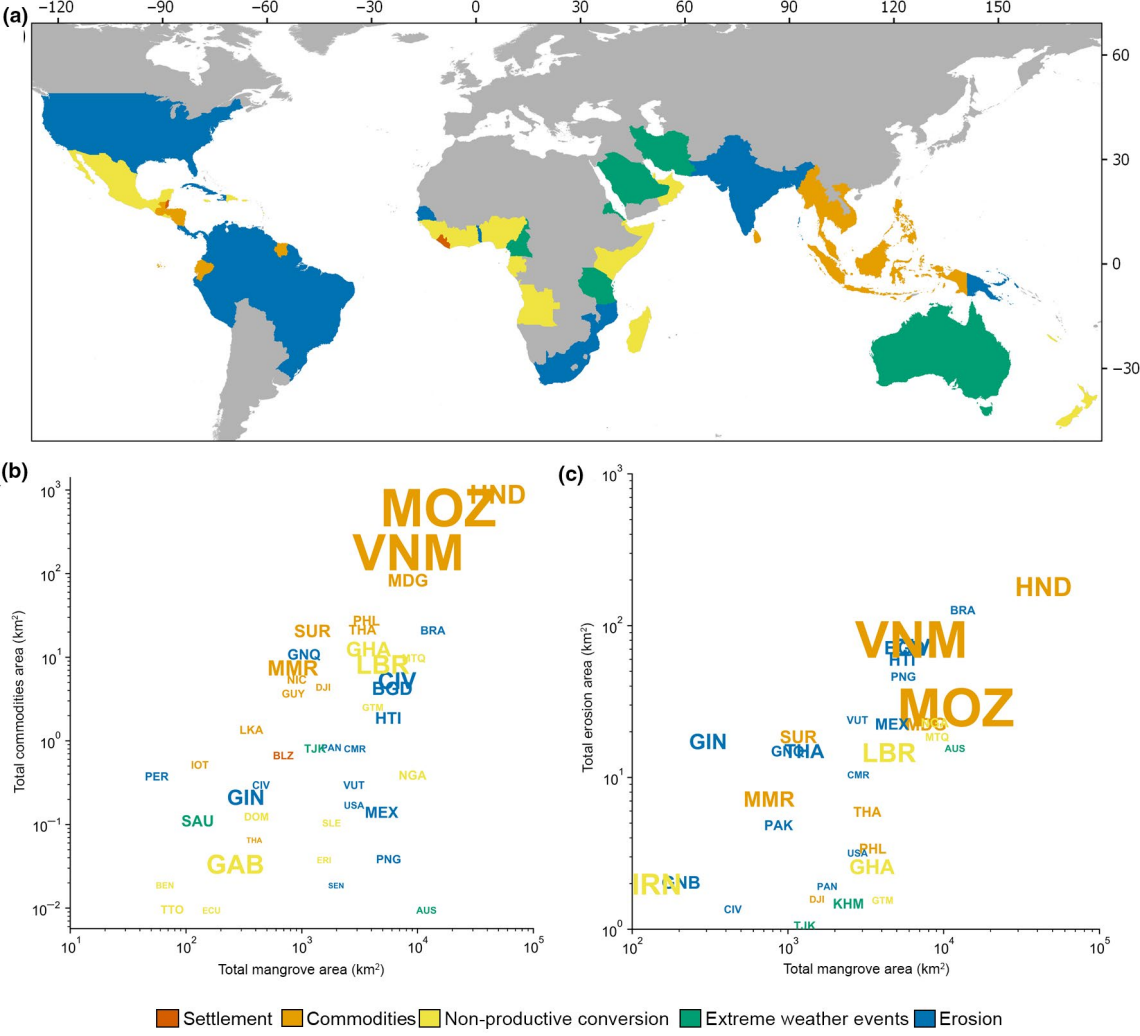
Country‐level trends in primary loss drivers. (a) Primary loss driver 2000–2016 by country. (b) National mangrove area lost to conversion to commodities as compared to the total mangrove area per country. (c) National mangrove area lost to erosion as compared to the total mangrove area per country. Countries are denoted by their ISO code (see Dataset S1 for details), font color relates to the country's primary driver, and font size is proportional to the total 2000–2016 percent loss by country. Percent loss ranges from 0% to 10% (see Dataset S1)

In mapping the distribution of natural and anthropogenic drivers over three distinct periods of the early 2000s, we reveal a significant human footprint on mangrove losses. These human impacts have been noted in previous studies (Richards & Friess, 2016), but have yet to be placed in a global context. Here we report that the majority of mangrove loss (62%) around the world since the start of the 21st century are a result of human impact along the coast (Dataset S1). Moreover, nearly half of all global mangrove losses were attributed to commodity‐based land use changes—a combination of rice, shrimp, and oil palm and other cultivation. Across each epoch, CM remained the primary proximate driver of mangrove change, but we measured a significant decline in the rate of direct anthropogenic loss from CM, ST, and NPC over the study period. As previous regional studies have shown, Southeast Asia contributes disproportionately to global anthropogenic losses, particularly as a result of conversion to CM (Hamilton & Casey, 2016; Richards & Friess, 2016; Figure 4a). Here we show that those regional CM hotspots account for 92% of all CM losses occurring in mangroves around the world and that these hotspots can occur within countries regardless of national mangrove inventory (Figure 4b).

**FIGURE 5:**
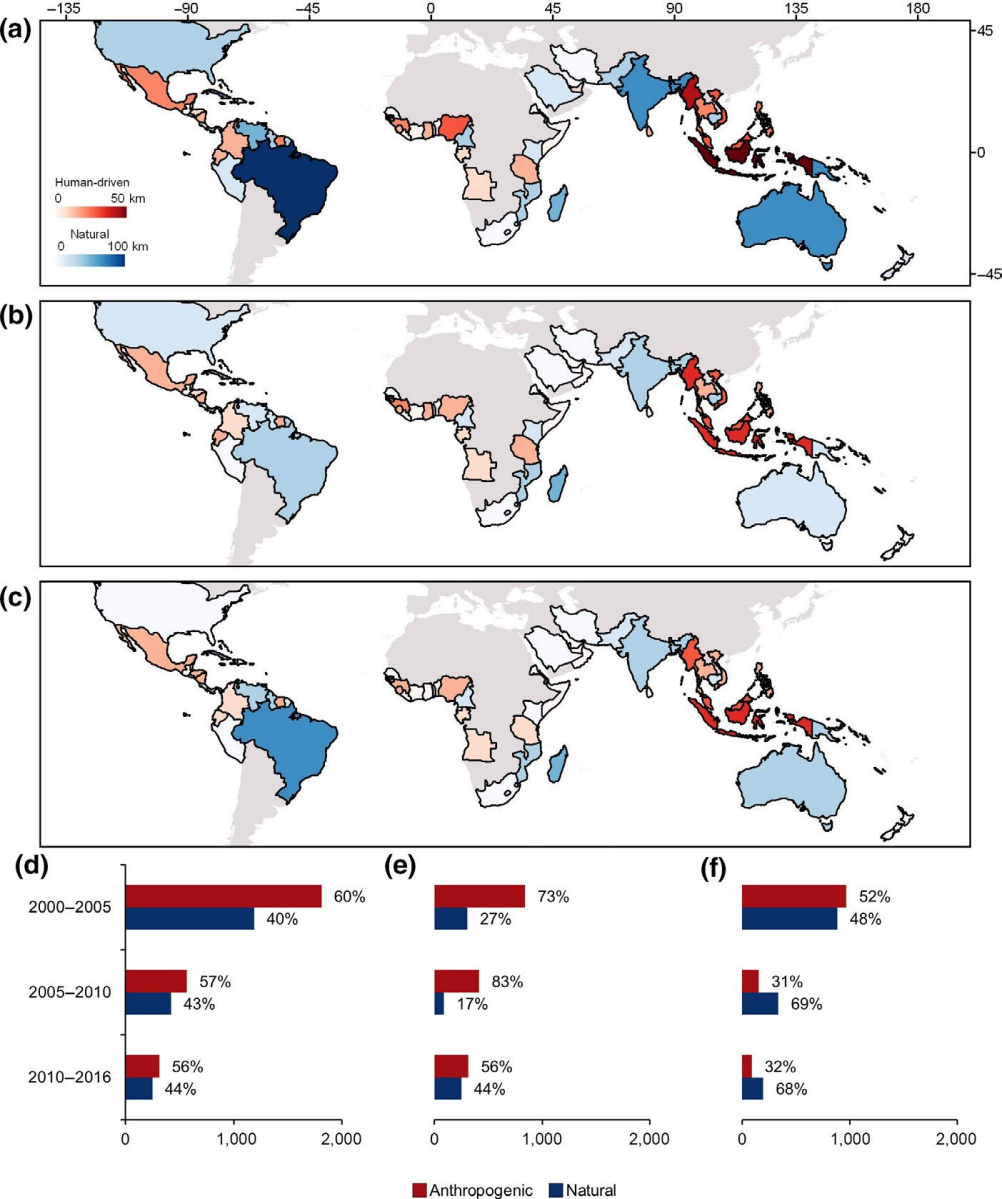
Anthropogenic and natural losses on the national and regional scale. The primary color of the country in parts (a–c) corresponds to the dominant category of mangrove loss on the national level (directly human‐driven or natural) per epoch, and the intensity of the color corresponds to the percentage of total loss driven by that driver category. (a) 2000–2005. (b) 2005–2010. (c) 2010–2016. (d) Global proportion of natural and anthropogenic loss per epoch. (e) Proportion of natural and anthropogenic loss per epoch in only Indonesia, Myanmar, Malaysia, the Philippines, Thailand, and Vietnam. (f) Proportion of natural and anthropogenic loss per epoch outside of the six Southeast Asian nations documented in part (e)

Natural losses also remained a significant factor contributing to global mangrove change. ER served as the second largest cause of mangrove loss, representing 27% of all losses. The total area of ER increased correspondingly with national mangrove inventory, remaining a prominent driver in even the primarily CM‐threatened nations of Southeast Asia (Figure 4c). However, excluding the six Southeast Asian nations with extensive CM‐driven loss, natural threats contribute the greatest area of global mangrove loss (Figure 5f). Shoreline ER then becomes the primary global loss driver, accounting for 43% of total (non‐SE Asia) losses (Dataset S1). With the exception of several anthropogenic hotspots, we identify that the majority of the world's mangroves are under the influence of ER and EWE.

### 4.2 Decline in global mangrove loss

Worldwide, the observed decline in human‐driven conversions may reflect a combination of site‐specific mangrove ecosystem service valuations and recent increase in interest for large‐scale restoration and conservation efforts. The value of mangrove ecosystem services have been known for centuries (Barbier et al., 2011; Primavera et al., n.d.), but until the early 21st century, mangrove restoration projects saw no significant changes in objectives, with silviculture as the dominant goal (Ellison, 2000). More recently, an increase in mangrove valuation studies (Vo, Kuenzer, Vo, Moder, & Oppelt, 2012) and a diversification of financial incentives aimed at effectively conserving and restoring mangrove ecosystems has occurred (Ahmed & Glaser, 2016; Herr & Landis, n.d.; Taillardat et al., 2018). Moreover, newfound awareness on the connections between mangroves and the reduction of economic damage and loss of life (Hochard, Hamilton, & Barbier, 2019) may have contributed to the reduction of loss during this period.

In some localized cases, the loss driver maps presented here demonstrate success in conservation‐associated reduction of further anthropogenic loss. In the Saloum Delta of Senegal, for instance, human activities resulted in only 0.1% of losses from 2000 to 2016, after decades of large‐scale restoration and conservation efforts resulting from previous exploitation in the region (Cormier‐Salem & Panfili, 2016; Hakimdavar et al., 2020). Likewise, in Southeast Asia, the 77% decline in CM may in part reflect a newfound regional emphasis on national policies encouraging aquaculture intensification over expansion(Friess et al. 2016).

However, conservation by law has often been historically ineffective in preventing continued anthropogenic loss, primarily due to inadequate monitoring or enforcement (Lee, Hamilton, Barbier, Primavera, & Lewis, 2019). We mapped human‐driven losses occurring in spite of national or international laws through the reclamation of mangroves for aquaculture in North Sulawesi, Indonesia (Figure 3c,d), the small‐scale cutting in the Rufiji Delta of Tanzania, and the rapid urban expansion on the border of the Can Gio Biosphere Reserve of Vietnam. Thus, while mangrove conservation and policy may have driven a portion of the decline in anthropogenic loss, declines in other regions could be a result of limitations in mangrove resources.

Observed declines in many of the CM hotspots of the 21st century could be a consequence of a lack of remaining mangroves viable for conversion to aquaculture or infrastructure. In Columbia, for instance, up to 20%–50% of the nation's mangroves have been designated as necessary to support shrimp farming activities (Larsson, Folke, & Kautsky, 1994). By 1993, Thailand converted 38%–65% of national mangrove area suitable for shrimp farms (Dierberg & Kiattisimkul, 1996). Mangrove areas were therefore facing widespread clearing for aquaculture in particular far before our study period, suggesting that the CM hotspots and declines in CM loss recorded here could be reaching local ecological capacity. With the observed historic losses and continued fragmentation (Bryan‐Brown et al., 2020), the decline in human exploitation is therefore expected within CM hotspots. This has the potential to place pressure on local municipalities to forgo conservation policies and convert viable mangrove resources in protected areas, such as the Tanjung Panjang Nature Reserve in Sulawesi, Indonesia (Figure 3c,d). Given their current status under observed decades of loss, knowledge on localized tipping points where insufficient forest area remains for conversion, as well as the location of viable areas that should be protected, is a critical area of future study.

While declines in human‐driven mangrove loss may mark a temporary stagnation in the extent of large‐scale losses worldwide, the continuation of pervasive naturally driven losses will compound the long‐term impacts of previous anthropogenic land‐use change, as barriers to landward migration of mangroves increase due to human ST (Rogers et al., 2019). The total area of natural losses declined from 624 km^2^ in *loss2005* to 249 km^2^ in *loss2016*, but the rate of decline in anthropogenic drivers exceeded the decline of natural drivers, suggesting that ocean related physical processes and EWE might ultimately become the dominant causes of global loss. Indeed, without the human influence from Southeast Asia, natural processes outpaced human impacts by nearly 2‐to‐1 in the final epoch (Figure 5f).

Total ER area declined between *loss2005* and *loss2016* by 60%, from 443 to 178 km^2^. Shoreline ER in mangroves ecosystems is the result of variability in sea level rise, rainfall, temperature, and wave activity (Asbridge, Lucas, Ticehurst, & Bunting, 2016; Gilman, Ellison, & Coleman, 2007; Sarwar & Woodroffe, 2013; Walcker et al., 2015). Upstream changes in water runoff and flow from dams and other structures can reduce sediment supply and therefore sedimentation to the coast, impacting shoreline processes (Lagomasino et al., 2019). The reduction in sediment supply to the coast, combined with ocean processes, may exacerbate shoreline retreat. However, for the scope of this study, ER was considered the physical removal of the shoreline due to ocean processes (e.g., sea level rise, waves, storms) and not a decrease in sedimentation. The decline in ER during the study periods could be a result of the downswing of multi‐annual variability in regional wave activity (Walcker et al., 2015). Though wave power has shown a measurable increase over the past half century, it has remained stable or shown a slight decrease during our study period (Reguero et al., 2019). Future fluctuations in wave activity and erosional processes will ultimately impact natural losses, and remain an area where additional research is needed.

Storms can have a significant effect on mangrove loss, both through ER and dieback (e.g., EWE; Cahoon et al., 2003; Radabaugh et al., 2019; Taillie et al., 2020). The decline in natural mangrove loss may be associated with a relatively low tropical cyclone landfall period from 2009 to 2016 and a lower accumulated cyclone energy, particularly for the Caribbean (Taillie et al., 2020). The intensity and frequency of tropical storms has increased (Bhatia et al., 2018; Sobel et al., 2016), thus monitoring the frequency and location of landfall necessitates further study to identify future trends in EWE. Despite the reduction in the natural rates of mangrove loss, the prevalence of these drivers suggests that future conservation policy should consider not only human‐driven conversions of the forests, but also natural stressors from oceanic processes that will emerge. For instance, the Southeast Asian Green Buffer policies(Friess et al. 2016) prohibiting the conversion of mangroves within a certain distance from the ocean may temporarily preserve the value of mangroves as storm surge barriers, but as the shoreline retreats (e.g., ER), the buffering distance between ocean and aquaculture ponds will be reduced in the coming years. The emergence of extreme weather‐driven mangrove losses is expected to continue in the future irrespective of land‐use policy, as stressors such as extreme events and sea level rise cause large‐scale disturbances regardless of protection status (Bryan‐Brown et al. 2020). Short‐term mangrove protection in regions immediately adjacent to eroding shorelines is necessary, but long‐term plans that account for coastal squeeze impacts as well as the effects of a changing climate are critical for maintaining mangrove ecosystem services.

### 4.3 Comparison to previous studies

Previous works at the regional scale have disaggregated mangrove loss into their proximate drivers of change. These efforts have largely centered on the intersection of the Mangrove Forests of the World (MFW; Giri et al., 2011) and the Global Forest Change (GFC; Hansen et al., 2013) datasets (Hamilton & Casey, 2016; Richards & Friess, 2016). We improve on these estimates by correcting for known alignment issues throughout certain regions within the MFW map (Gandhi & Jones, 2019). Similarly, we overcome the water masking issues with the GFC loss layer (Lagomasino et al., 2019) to better capture ER along the seaward margin and other flooding conditions. The GFC dataset also captures considerable temporary loss in regions that could recover quickly after cyclones and other EWE (Taillie et al., 2020). Differences in methodology, classification types, and time period make a direct comparison difficult (Table S1), but ultimately the data presented here support previous findings on the dominance of commodity‐driven loss in Southeast Asia. We now show that these commodity‐driven losses are, in fact, the majority cause of mangrove loss worldwide.

In a recent global analysis of drivers of mangrove loss, ER was identified as a major influence on mangrove forests (Thomas et al., 2017). Indeed, ER was among the dominant drivers of mangrove loss across all continents as measured by the single occurrence of each driver within 1°×1° geographic grid cells. Here we support these findings by quantifying the continued prevalence of ER as substantial global threat. We show that ER losses contribute 27% of global losses and occur in nearly all mangrove‐holding nations. Moreover, we estimate that without the dominant influence of CM in select regions, ER would be the primary cause of global mangrove loss (Figure 5).

### 4.4 Limitations of change maps

Though this study provides the first global quantitative estimates of natural and anthropogenic mangrove loss drivers, the use of previously established datasets such as global agriculture or settlement layers propagates error into the disaggregation of each driver class. For example, in separating mangrove dieback due to natural and anthropogenic factors (EWE and NPC, respectively), only the presence/absence of roads, ST, or CM was used as a determinant of the likelihood of human intervention. The identification of anthropogenically influenced patches of dieback did not account for other forms of infrastructure such as dams, with the remainder of conversions being classified as a result of EWE. While the 73% and 68% accuracy, respectively, of these classes justifies this method, using a compilation of several extreme weather datasets would be beneficial toward identifying the proportion of these natural diebacks that truly result from weather events. Furthermore, the use of sea level rise and land subsidence estimates and projections would enable the identification of pixels of ER influenced directly by changing sea levels, moving toward a quantitative global estimate of the impact of climate change on mangrove loss. Our current mangrove loss maps are based solely on proximal‐based anthropogenic and natural threats.

## 5 IMPLICATIONS AND CONCLUSIONS

Global initiatives such as the Global Mangrove Alliance recently set the goal of increasing global mangrove area by 20% by 2030, inspiring widespread restoration and rehabilitation projects worldwide (Friess et al. 2020). In developing high‐resolution and thus locally relevant loss driver maps, we enable conservation and restoration plans to modify their adaptive management strategies on the basis of the intersections of various stressors. This study's global‐scale quantification of the proximate drivers of mangrove loss will be coordinated with other global‐scale mangrove studies, providing the mangrove stakeholders around the world with transparent and open data to in order to increase the update and impact of large‐scale mangrove research and conservation (Worthington et al., 2020). Conservation and restoration plans will differ significantly based on the type of mangrove loss (Lewis, 2005).

Our findings suggest that human activity has been a dominant cause of mangrove forest loss, but its impact has decreased since 2000. We observe the emergence of natural drivers as the primary causes of modern mangrove loss, as a consequence of both a lack of available viable mangrove forest and the emergence of effective conservation initiatives. The future sustainability of mangrove forests must consider the severity and intensification of extreme weather and ocean processes as increasingly important drivers of global losses. By disaggregating individual loss drivers, this work can aid restoration efforts by revealing ongoing stressors past and present within a specified region, making management efforts more informed and effective. Regardless of the current level of direct human intervention in the forest, the intersection of existing anthropogenic and future climatic losses must be considered when enacting future ecosystem valuation and conservation on an increasingly human‐dominated planet.

## AUTHOR CONTRIBUTION

L.G., D.L., and T.F. conceived and designed the study. D.L. carried out the loss extent mapping. L.G. led the land cover change and loss driver modeling, interpretation of data, and generation of results, with assistance from D.L and N.T. The writing of the paper was led by L.G. with contribution from D.L., N.T., and T.F.

## Supporting information

Supplementary Material

Supplementary Material

## Data Availability

All loss driver, land cover change, and loss extent maps from this study are available on the Oak Ridge National Data Archive (ORNL DAAC) as GEOTIFF files (https://doi.org/10.3334/ORNLDAAC/1768). An interactive mapping portal as well as additional data visualization options are available at mangrovelossdrivers.app.
