# Supplementary material for: Global declines in human‐driven mangrove loss
Source: Glob Chang Biol. 2020 Aug 3;26(10):5844–55. doi: 10.1111/gcb.15275 (PMC7540710; doi:10.1111/gcb.15275)
Supplement: Supplementary file 1 — Supplementary Material [file GCB-26-5844-s001.pdf]

Supplementary Methods

Landsat Imagery Preprocessing.

All Landsat imagery was preprocessed for surface reflectance using the Landsat Ecosystem Disturbance Adaptive Processing System (LEDAPS) for Landsat 5 TM and 7 ETM+ (*43*), and Landsat Surface Reflectance Code (LASRC) for Landsat 8 Operational Land Imager (OLI) (*44*). A cloud mask, CFMask algorithm, was derived for each image and used to remove cloudy pixels (*45*). The Surface Reflectance Tier 1 product in Google Earth Engine was used to develop a time series dataset that spanned 1998 to 2016. Landsat 5 TM, 7 ETM+, and 8 OLI were first harmonized to correct for different sensor specifications (*46*). After the harmonization process, the Normalized Difference Vegetation Index (NDVI) was calculated for each image in the collection. We limited the extent of the imagery to regions that were within two kilometers of mangroves designated by the GMFD map for 2000 (*38*).

Global Mangrove Forest Extent Definition.

The use of the GMFD dataset to mask all mangrove regions in the year 2000 may have caused error in both the exclusion of relevant mangrove areas (particularly in small island nations (*47*)), and calculation of percent loss per mangrove-holding country. All mangrove loss maps were masked using a modified vector file with corrections to the original GMFD raster found on Google Earth Engine, due to significant geographic offset in the extent of mangroves in western South America, East Africa, and Southeast Asia in particular. The total areal extent of mangroves from this vector file is 160,086 km^2, nearly 20% higher than the original total mangrove area value recorded in the GMFD (*38*). The global mangrove area calculated from the original raster file found on Google Earth Engine was also 10% higher than the reported value of 1,378 km^2. We used the corrected GMFD vector dataset to mask global mangrove extent because

of the better overlap with coastal margins, and thus we report the national and continental loss percentage values according to the vector-derived total mangrove area. However, we also provide a range of loss rates according to the original GMFD raster file in Supplemental Dataset 1.

Loss Driver Decision Trees.

Within the initial water land cover class, pixels were separated into the final loss driver class of either erosion or aquaculture. Erosion was defined as loss intersecting a river or coastline connected to the open ocean, as derived from the 30-m resolution Global Forest Change 2016 (*36*) water mask and the JRC Global Surface Water 2016 occurrence layer (*48*). In regions of high aquaculture density, as visually classified in each of the 1168 1° x 1° grid cells used in initial sampling, a vector-based spatial join approach was used to identify water pixels that intersected either the modified Global Forest Change or JRC water masks, where vectors intersecting both masks were classified as erosion. All other non-erosion vectors within high-density aquaculture grid cells were assigned to the aquaculture class, later to be merged with the agriculture class to form a general commodity-driven loss category. In low aquaculture density tiles, an object-based approach was used to identify clusters of water pixels adjacent to the modified Global Forest Change water mask, which were classified as erosion. All non-connected water clusters were classified as dieback. The differentiation in techniques for erosional separation according to the density of aquaculture in the tile resulted from frequent confusion between erosion and aquaculture in high density aquaculture regions. Since all water cover pixels contiguous to the water mask—regardless of whether the mask stretched inland from river channels and oceans—were classified as erosion, large clusters of aquaculture were misclassified as erosion. Moreover, confusion between extreme weather events and erosion occurred in low

density areas, where the water mask did not reach the coast and thus missed erosion pixels. We thus implemented the vector-based join approach in high density regions because of its ability to better separate connected aquaculture and erosion clusters, while the object-based connected pixels approach more easily captured adjacent erosion and water.

Most dry soil-converted pixels occurred in impervious surfaces or dry sandy soils near coastal water bodies (fig. S7c-d). Human settlement-driven conversion to impervious surfaces was identified using the Global Human Settlements Layer (GHSL) low density and high-density settlement layers, while all other pixels were assumed to be dry sandy-to-muddy soils and were assigned to the general dieback class, later to be further separated into human or extreme weather events. Likewise, visual observation of pixels converted to wet soil confirmed that the major driver of wet soil conversion was agriculture, though some land cover changes resulted from degradation to mudflats. As such, the Global Food Security-support Analysis Data Cropland Extent 30-m (GFSAD-30) layer (*49–55*) was used to mask all agricultural areas, with the remainder of the wet soil-converted pixels assigned to the dieback class.

The separation between directly human-driven non-productive conversion and dieback from extreme weather events remained a challenge due to the often-simultaneous anthropogenic and natural factors affecting forest degradation at varying scales. In this study, we sought to solely identify losses that had a high likelihood of direct human intervention, in order to distinguish these regions from other areas without anthropogenic influences. We define dieback as conversion from mangrove to an unused land type—one that has not been converted to commodities or settlement, or naturally transitioned to open water or mudflat through processes such as erosion. All non-productive conversions and extreme weather event-driven losses were considered diebacks. In order to identify the potential extent of human intervention, we derived a

“human influence” layer using a 5.5 km buffer around the GRIP-4 global roads dataset (*56*) and the GHSL human settlement dataset (*57*). The additional buffer helped to capture mangrove losses associated with small-scale cutting near roads or settlements and hydrologic changes resulting from built environments. Any loss that occurred outside of the human influence buffer was assigned to the extreme weather events class on the basis that losses occurring at a significant distance from known human settlements likely saw little direct anthropogenic influence, and the dieback was therefore assumed to result from an extreme event such as a drought or cyclone.

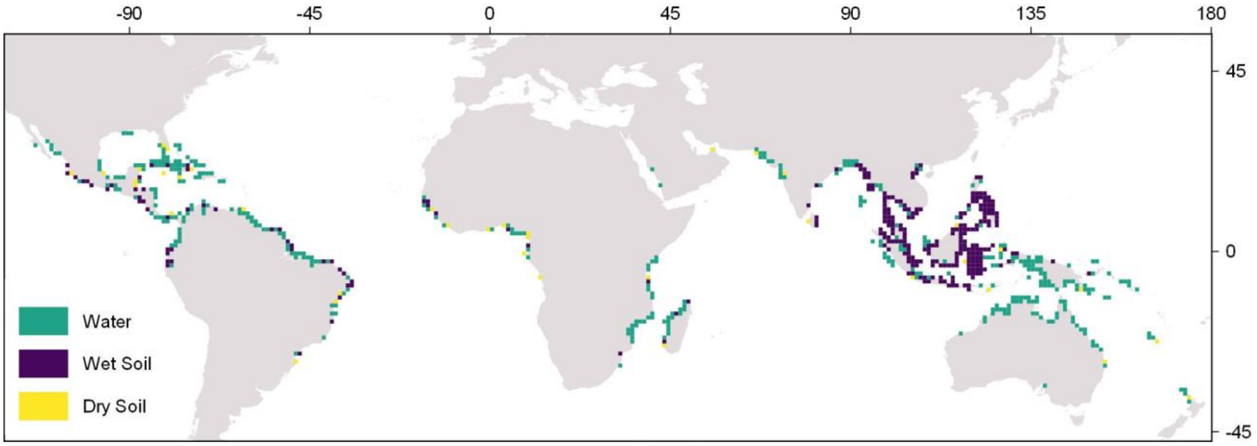

Fig. S1| Distribution of global mangrove landcover changes. The primary Random Forest-classified landcover change per 1° x 1° grid cell from 2000 to 2016.

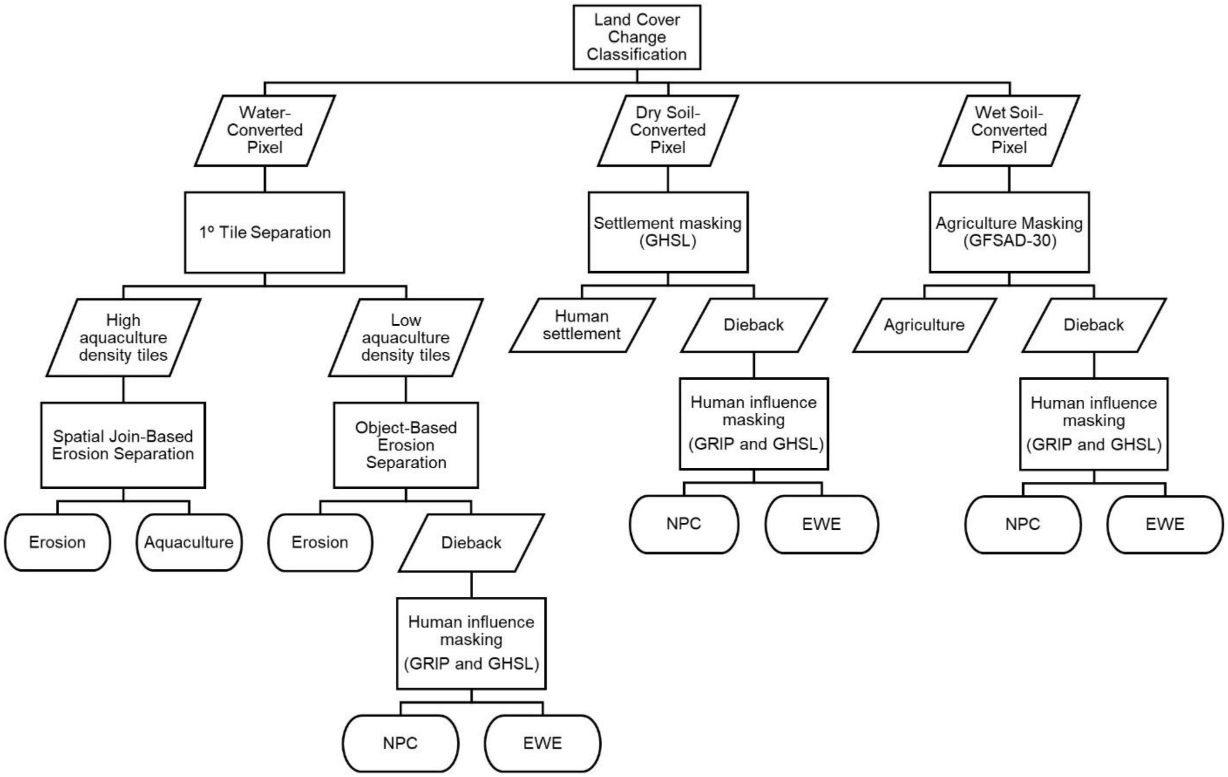

Fig. S2| Decision-tree framework for loss driver mapping. The initial landcover change classes of water, wet soil, and dry soil provided the basis for a series of decisions that used global-scale datasets to classify the ultimate land use change. ECE represents loss by extreme weather events, and NPC represents loss by non-productive conversion.

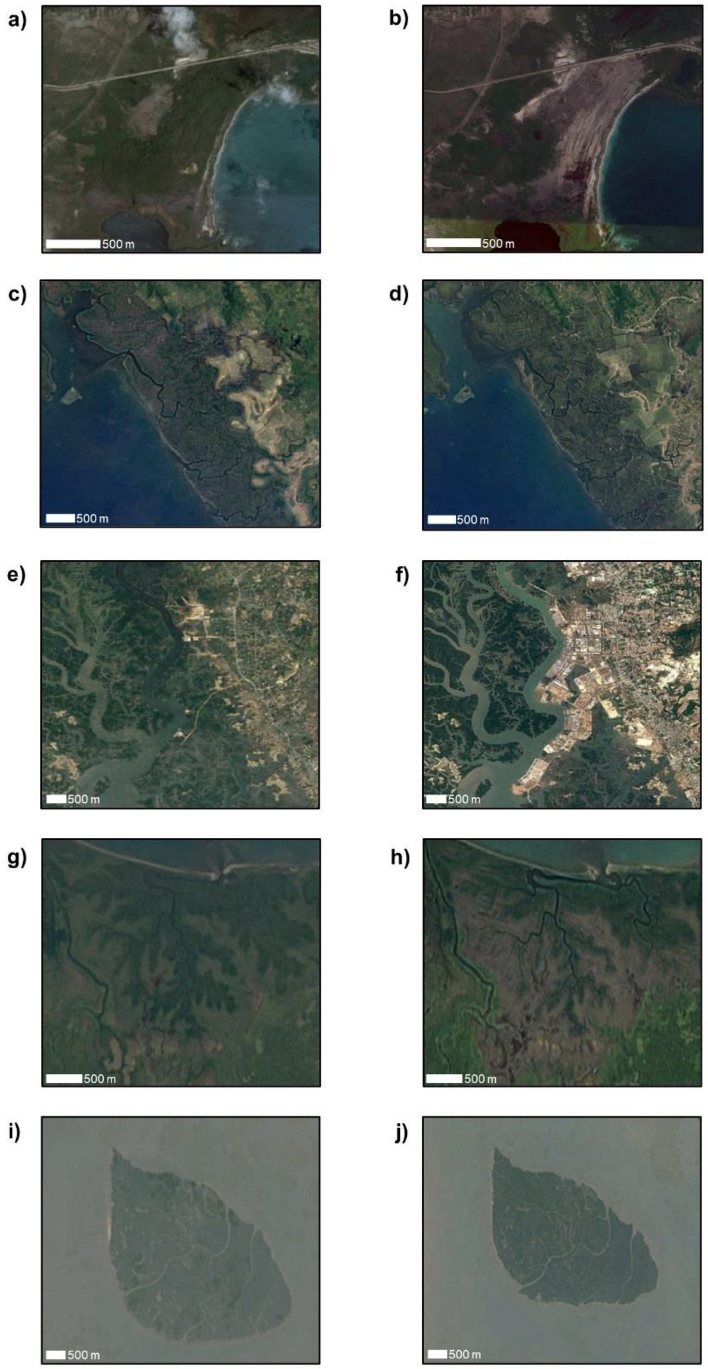

Fig. S3| Examples of loss driver classes. The left image in each pair shows Google Earth imagery from approximately 2000, and the right image shows Google Earth imagery from approximately 2016. (**a-b)** Non-productive conversion due to hydrologic change from Rocky Point Main Road near Colon Bay, Jamaica. (**c-d)** Conversion to aquaculture in Tanjung Panjang

Nature Preserve in Sulawesi, Indonesia. (**e-f)** Conversion to settlement in Ho Chi Minh City, Vietnam. (**g-h)** Dieback from extreme weather events (Cyclone Guba) in Oro Province, Papua New Guinea. (**i-j)** Erosion in the Bangladesh Sundarbans.

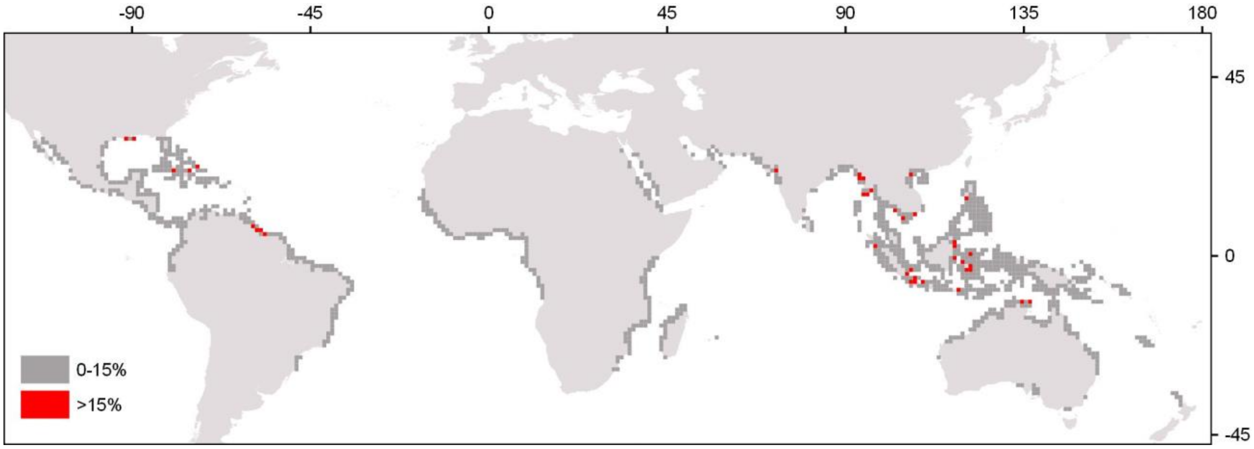

Fig. S4| Global mangrove loss hotspots. Total loss percent was calculated per 1° x 1° grid cell from 2000 to 2016 from the Giri *et al.* (*38*) mangrove vector dataset. Hotspots were designated as tiles with greater than 15% mangrove loss from 2000-2016.

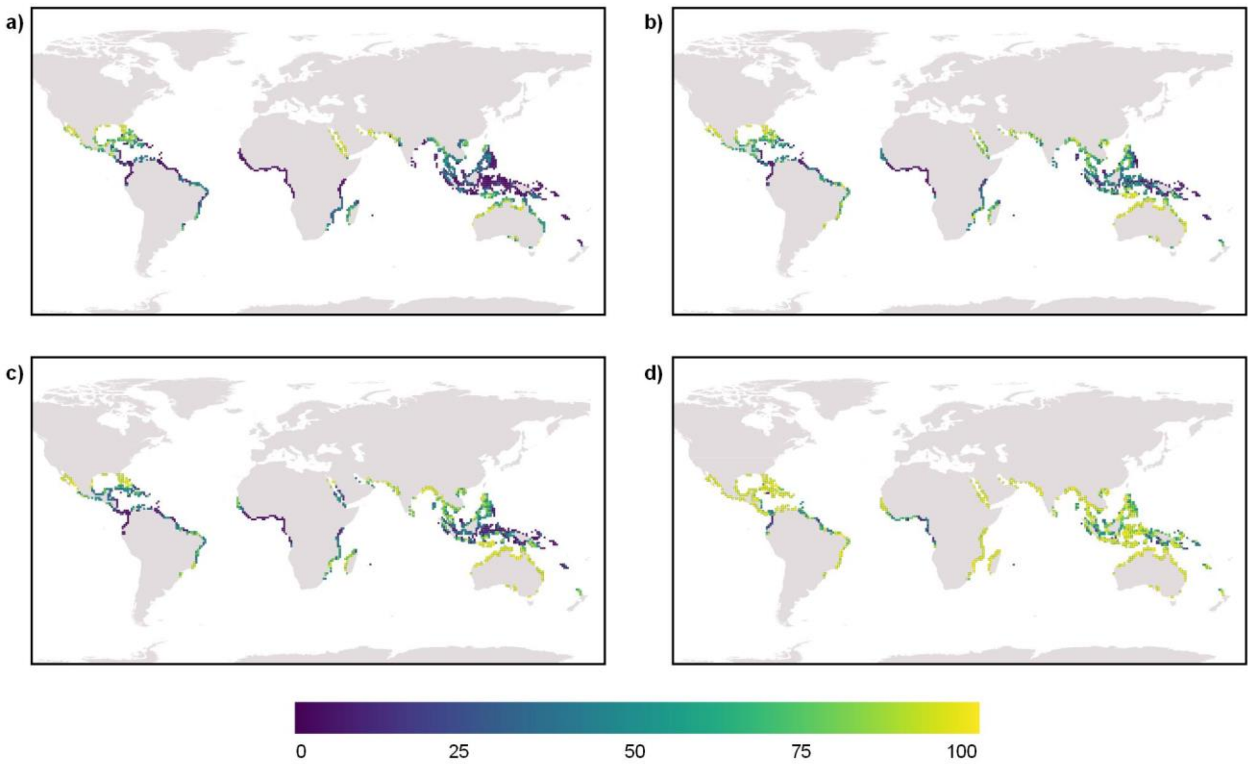

Fig. S5| Count of Landsat images used in loss extent mapping. The number of Landsat images used per 1° x 1° grid cell in the reference and series periods of the global NDVI anomalies: (**a**) Reference period: 1998-2001. (**b**) Series period: 2000-2005. (**c**) Series period: 2005-2010. (**d**) Series period: 2010-2016.

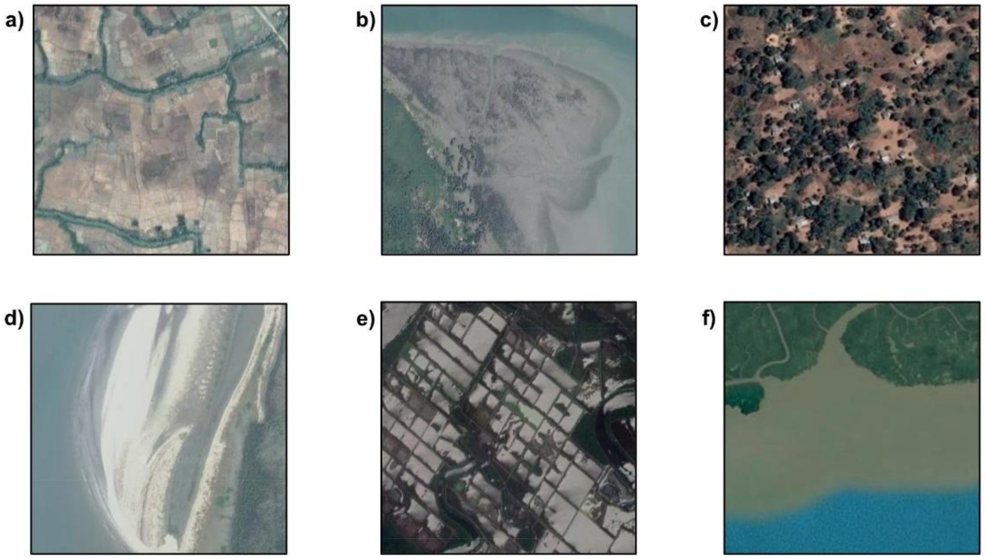

Fig. S6| Representative samples of Google Earth imagery used in Random Forest classifier. Landcover change points (n=2,669) were sampled among the wet soil, water, and dry soil classes in 1,168 1º x 1º grid cells containing mangrove forest in the year 2000. Each panel shows an example of a sampled landcover type eventually consolidated into one of the three major landcover classes: (**a)** Agriculture and (**b)** Mudflat were considered wet soil; (**c)** Settlement and (**d)** Sand were considered dry soil; and (**e)** Aquaculture and (**f)** Rivers and oceans were considered water.

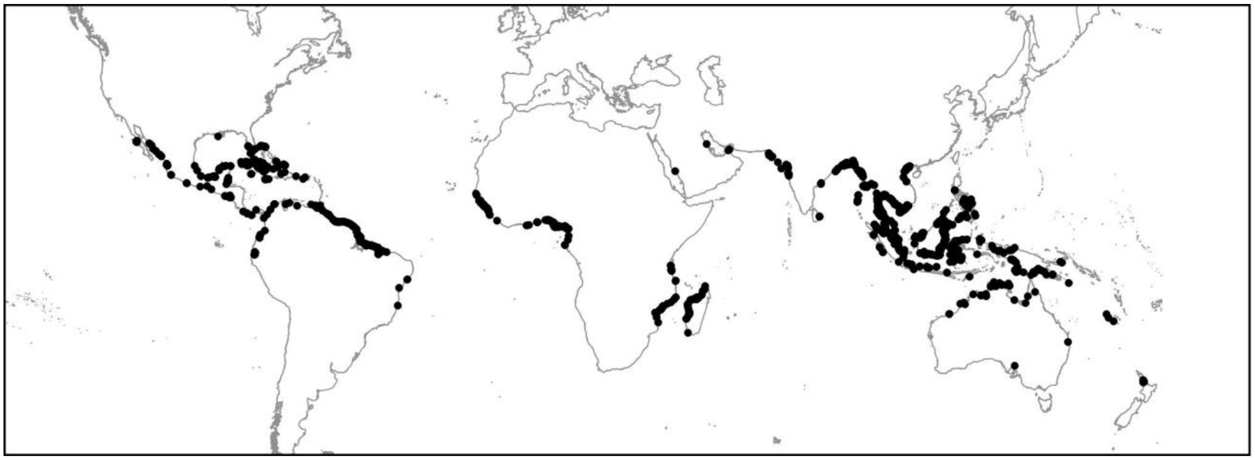

Fig. S7| Distribution of randomly sampled points for accuracy assessment. Randomly sampled points (n=2,476) were stratified between the five loss driver classes (commodities, erosion, non-productive conversion, extreme weather events, and settlement) according to the global area lost to each driver. All points were assessed using recent Google Earth imagery using a QGIS accuracy assessment plugin.

Table S1| Comparison of this study with Richards & Friess (*5*) **estimates of mangrove area, deforestation area, and annual deforestation rate in Southeast Asia.** To facilitate comparison between the two datasets, "deforestation" in the Richards and Friess dataset was considered anthropogenic loss (the combination of commodities, settlement, and non-productive conversion) in this manuscript. Total Southeast Asian mangrove area, deforestation area, and annual deforestation rate were recalculated for the Richards and Friess dataset without Timor-Leste, which was not included in this manuscript's analysis. Annual deforestation rates were calculated by dividing the total area of loss over the study period by the number of years in the study period.

Country	Total mangrove Area 2000 (ha), Richards and Friess	Total mangrove Area 2000 (ha), Goldberg et al.	Mangrove Deforestation 2000-2012 (ha), Richards and Friess	Mangrove Deforestation 2000-2016 (ha), Goldberg et al.	Annual rate of Mangrove Deforestation 2000-2012, Richards and Friess	Annual Rate of Mangrove Deforestation 2000-2016, Goldberg et al.
Indonesia	2,788,683	3,030,999	60,906	85,336	0.2%	0.2%
Myanmar	502,466	591,955	27,957	44,485	0.5%	0.5%
Malaysia	557,805	623,930	18,836	10,391	0.3%	0.1%
Thailand	245,179	293,332	3,504	2,880	0.1%	0.1%
Philippines	257,575	318,863	1,423	3,365	0.0%	0.1%
Cambodia	47,563	55,848	1,218	754	0.2%	0.1%
Vietnam	215,154	257,983	531	13,915	0.0%	0.3%
Brunei	11,054	12,960	48	61	0.0%	0.0%
Singapore	583	756.94	0	0	0.0%	0.0%
Southeast Asia	4,626,062	5,186,626	114,423	161,189	0.2%	0.2%

Table S2| Error matrices for global loss driver accuracy assessment.

a)

	Commodities	ECE	Settlement	Erosion	NPC	Other	User
Commodities	975	6	20	38	57	8	1104
EWE	21	181	3	41	11	9	266
Settlement	11	1	155	7	26	0	200
Erosion	51	41	0	426	2	2	522
NPC	37	3	4	52	280	8	384
Other	0	0	0	0	0	0	0
Producer	1095	232	182	564	376	27	2017

b)

	Commodities	ECE	Settlement	Erosion	NPC	Other	User (%)
Commodities	39.38	0.24	0.81	1.53	2.3	0.32	88.32
EWE	0.85	7.31	0.12	1.66	0.44	0.36	68.05
Settlement	0.44	0.04	6.26	0.28	1.05	0	77.5
Erosion	2.06	1.66	0	17.21	0.08	0.08	81.61
NPC	1.49	0.12	0.16	2.1	11.31	0.32	72.92
Other	0	0	0	0	0	0	0
Producer (%)	89.04	78.02	85.16	75.53	74.47	0	81.46

c)

	Area Estimate (km^2)	Class Area Uncertainty (km^2)
Commodities	1596.21	±41.70
ECE	350.77	±31.36
Erosion	912.19	±41.09
Settlement	96.19	±14.99
NPC	397.64	±29.38

(a) Error matrix of all sample counts (n=2,476). (**b)** Error matrix of sample percentage accuracies. (**c)** Adjusted class areas based on error matrix. ECE represents loss by extreme weather events, and NPC represents loss by non-productive conversion.
